# Surface Structure Dependent Electrocatalytic Activity of Co_3_O_4_ Anchored on Graphene Sheets toward Oxygen Reduction Reaction

**DOI:** 10.1038/srep02300

**Published:** 2013-07-29

**Authors:** Junwu Xiao, Qin Kuang, Shihe Yang, Fei Xiao, Shuai Wang, Lin Guo

**Affiliations:** 1 Department of Chemistry, William Mong Institute of Nano Science and Technology, The Hong Kong University of Science and Technology, Clear Water Bay, Kowloon, Hong Kong; 2 Department of Chemistry and Chemical Engineering, Hubei Key Laboratory of Matieral Chemistry and Service Failure, Key Laboratory for Large-Format Battery Materials and System, Ministry of Education, Huazhong University of Science & Technology, Wuhan, PR China; 3 School of Chemistry & Environment, Beihang University, Beijing, PR China

## Abstract

Catalytic activity is primarily a surface phenomenon, however, little is known about Co_3_O_4_ nanocrystals in terms of the relationship between the oxygen reduction reaction (ORR) catalytic activity and surface structure, especially when dispersed on a highly conducting support to improve the electrical conductivity and so to enhance the catalytic activity. Herein, we report a controllable synthesis of Co_3_O_4_ nanorods (NR), nanocubes (NC) and nano-octahedrons (OC) with the different exposed nanocrystalline surfaces ({110}, {100}, and {111}), uniformly anchored on graphene sheets, which has allowed us to investigate the effects of the surface structure on the ORR activity. Results show that the catalytically active sites for ORR should be the surface Co^2+^ ions, whereas the surface Co^3+^ ions catalyze CO oxidation, and the catalytic ability is closely related to the density of the catalytically active sites. These results underscore the importance of morphological control in the design of highly efficient ORR catalysts.

The advent of nanotechnology and the ability to synthesize a marvelous panoply of nanocrystals have breathed a new life to the catalysis science^1^,^2^,^3^,^4^,^5^. The notion that catalysts are necessarily nanomaterials is rooted in the importance of surface in activating chemical bonds. Although there have been numerous reports on the catalytic activities of nanomaterials, detailed understanding of how surface structure affects catalytic performance is still lacking. There is therefore a need to systematically study the catalytic activity as a function of nanocrystalline morphology other than the size since the surface structure is tunable by varying the morphology. The prerequisite is the selective synthesis of differently shaped nanocrystal catalysts with uniform crystal surfaces, preferably dispersed on a supporting substrate.

The spinel type Co_3_O_4_, in which the Co^2+^ and Co^3+^ ions occupy the tetrahedral and octahedral sites, respectively^6^, is known to be a promising catalytic material^7^,^8^,^9^,^10^,^11^,^12^. It has been reported that different morphologies of Co_3_O_4_ nanocrystals have a direct bearing on their catalytic activities for CO oxidation. For example, the {110} faces of Co_3_O_4_ nanocrystals have a higher catalytic activity for CO oxidation than {100} and {111}, because of the more abundant catalytically active Co^3+^ sites on the former^12^. For the CH_4_ combustion, however, the catalytic activity of the nanocrystalline surfaces was found to be in the order of {112} > {011} ≫ {001}, depending instead on the surface energy^13^. In the main, the catalytic activity of a given catalyst is therefore determined by the nature of adsorption/activation/desorption of the reactants and products on the catalytically active sites^12^,^13^,^14^,^15^.

The spinel-type Co_3_O_4_ nanocrystals are also a potential alternate for the high cost Pt and its alloys to catalyze the oxygen reduction reaction (ORR), a critical reaction which underlies a battery of renewable-energy technologies such as fuel cell. To our knowledge, however, no study has been reported on the correlation between the shape and the ORR catalytic activities of Co_3_O_4_ nanocrystals. Such a study requires anchoring the Co_3_O_4_ nanocrystals onto a substrate, which is preferably conductive and thus can enhance the ORR activity and stabilize the catalyst system. As a relatively new class of carbon-based nanomaterials, graphene and carbon nanotube (CNT) have high electrical conductivity, large surface area, high mechanical strength, and structural flexibility, making them ideal substrates for supporting such nanocrystal catalysts. Indeed, graphene and CNT supported Co-based electro-catalysts have already been used for ORR with improved catalytic activity and stability^16^,^17^,^18^,^19^. However, shape-controllable synthesis of Co_3_O_4_ nanocrystals on graphene and CNT as composites is still an unmet challenge.

In this paper, we report the controllable synthesis of Co_3_O_4_ nanorods, nanocubes and nano-octahedrons with difference exposed surfaces uniformly immobilized in situ on graphene sheets. This series of nanocrystals showed much enhanced ORR catalytic activity when dispersed on graphene. More significantly, the quantitative catalytic activity depends on the detailed nanocrystalline morphology and thus the surface structure of the nanocrystals, namely, {111} > {100} > {110}, pointing to the Co^2+^ ions as the ORR active sites.

## Results

### Shape-selective synthesis of Co_3_O_4_ nanocrystals

Detailed procedures for the synthesis of Co_3_O_4_ nanoparticles (NP), nanorods (NR), nanocubes (NC) and nano-octahedrons (OC) on the surface of reduced graphene oxides (RGO) have been given in the experimental section, and are here illustrated in Scheme 1. The crystalline phases of these nanocomposites were ascertained by XRD patterns (Figure SI-1), with the help of the standard crystal structure of Co_3_O_4_ (JCPDS 65-3103). Co_3_O_4_ NP around 10 nm across were formed by thermally decomposing the precursors nucleated from the supersaturated metal bicarbonate solution accompanied by the slow release of CO_2_ (Figure SI-2), as we reported previously^20^,^21^,^22^.

**Figure 1:**
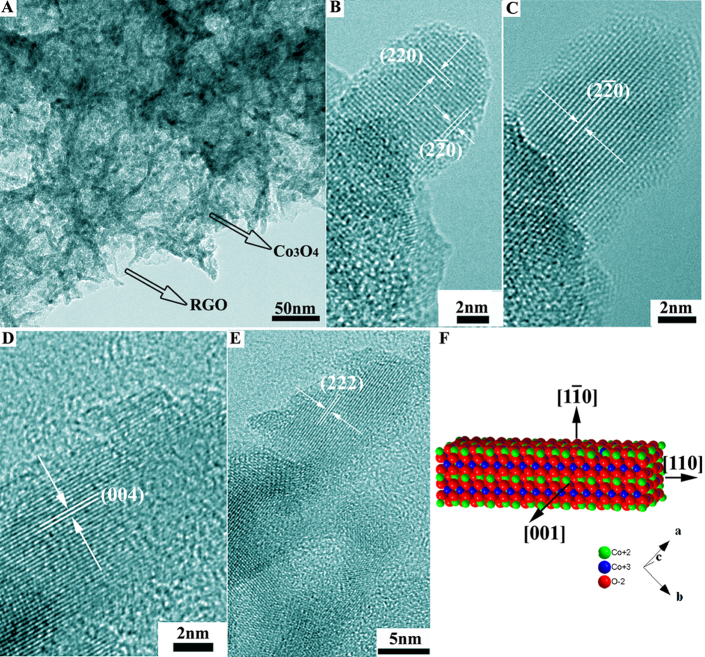
Co_3_O_4_ nanorods anchored on the RGO sheets. Low magnification TEM image (A); HRTEM images viewed along the (B, C) [001] and (D, E) [1–10] axes; (F) Schematic illustration of the nanorod morphology highlighting the exposed surfaces. Note: [uvw] is a crystal axis index, (hkl) is a crystal plane index.

Presumably, through the Ostwald ripening process, the initially nucleated precursors were transformed into cobalt carbonate hydroxide (Co(CO_3_)_0.5_OH) nanorods with a length of several hundred nanometers and a diameter of ~10 nm (Figure SI-3)^21^. The subsequent calcination process caused a spontaneous transformation into the Co_3_O_4_ NR with the overall morphology conserved. Figure 1A shows a typical low-magnification transmission electron microscopy (TEM) image of the synthesized Co_3_O_4_ NR, densely distributed on the surface of RGO sheets. We further examined the crystallographic nature of the individual Co_3_O_4_ NR through high resolution TEM (HRTEM) observations. Shown in Figure 1B is a section of the Co_3_O_4_ NR observed in the [001] orientation, which extends along the [110] direction. The side walls are parallel to the (2–20) plane (Figure 1B, C). When the nanorod was titled to the [1–10] zone axis, the (004) side planes and the (222) crystal planes could also be clearly observed (Figure 1D, E). On the basis of these results, the nanorod morphology can be approximately laid out as shown in Figure 1F: the nanorod assumes its axial direction along [110] and is bounded by the side planes of {001} and {1–10}. Similar Co_3_O_4_ NR were prepared previously by the calcination of cobalt carbonate hydroxide nanorod precursors obtained by the precipitation of cobalt acetate and sodium carbonate in ethylene glycol, and used for low temperature catalytic CO oxidation^12^.

**Figure 2:**
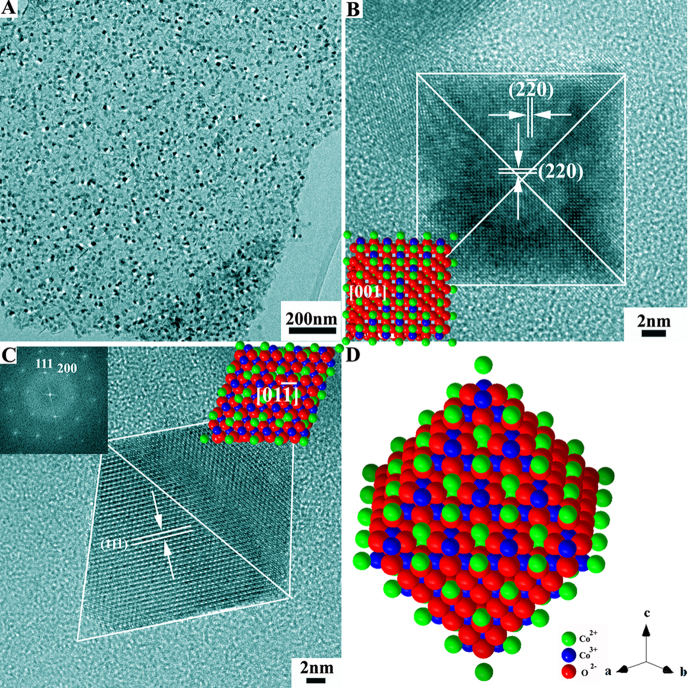
Co_3_O_4_ nano-octahedrons anchored on the RGO sheets. (A) Low magnification TEM image; (B) HRTEM image along the [001] direction; (C) HRTEM image along the [01-1] direction; and (D) Schematic illustration of a nano-octahedron bounded by the eight {111} surfaces.

When H_2_O_2_ was added, during the Ostwald ripening process, the cobalt carbonate hydroxide (Co(CO_3_)_0.5_OH•0.11H_2_O) nanorods were gradually transformed into Co_3_O_4 _nanocrystals, as seen from SEM images of the intermediates (Figure SI-4). After complete transformation, well-defined Co_3_O_4_ OC were uniformly dispersed on the surface of graphene sheets (Figure 2A). To reveal the exposed surfaces, we refer to the TEM images in Figure 2. According to the HRTEM image of a single Co_3_O_4_ OC particle taken along the [001] direction (Figure 2B), the two-dimensional (2D) projection appears to be square (see the model octahedron in the bottom left inset in Figure 2B). By tilting the Co_3_O_4_ OC to the [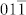] zone axis, the 2D projection of the corresponding TEM image (Figure 2C) becomes diamond-like. These results are consistent with the octahedron morphology of the Co_3_O_4_ nanocrystals anchored on the surface of RGO sheets with eight exposed {111} surfaces (see the model structure in Figure 2D).

**Figure 3:**
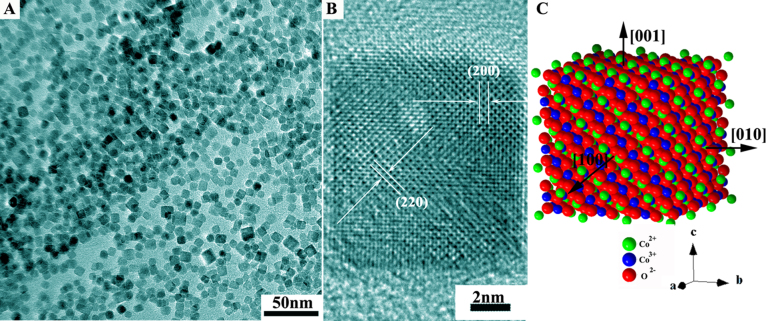
Co_3_O_4_ nanocubes grown on the RGO sheets. (A) Low magnification TEM image; (B) HRTEM image; and (C) Schematic illustration of the nanocube morphology with the six exposed {100} surfaces.

Finally, Co_3_O_4_ NC were formed on the surface of RGO sheets by co-precipitation of Co^2+^ and ammonia followed by hydrothermal treatment in the presence of H_2_O_2_. TEM observations revealed that the as-formed Co_3_O_4_ nanocrystals have a perfect cubic morphology and a uniform crystallite size of about 10 nm (Figure 3A). Excellent crystallinity of the Co_3_O_4_ NC was confirmed by the HRTEM image in Figure 3B. The lattice fringes of *d*_200 _(0.418 nm) and *d*_220_ (0.285 nm) of Co_3_O_4_ are clearly observed from the [001] direction, indicating the nanocubes bounded by the (001) facets (see the constructed nanocube model in Figure 3C).

The composition of the prepared cobalt oxides was investigated by XPS spectra, which are shown in Figure SI-5A. The Co 2p spectra all show a doublet consisting of a low energy band (Co 2p_3/2_ at 780. 6 eV) and a high energy band (Co 2p_1/2_ at 796.0 eV) for the Co_3_O_4_ NP, NR, NC and OC, in agreement with the standard spectra of Co_3_O_4_^23^,^24^. The energy difference between the peak of Co 2p_3/2_ and 2p_1/2_ splitting is approximately 15 eV, indicating the presence of both Co^2+^/Co^3+^ species in the cobalt oxides samples^23^,^24^,^25^. The RGO contents in the composites were obtained from the TGA curves (Figure SI-5B), and calculated based on the weight loss below 400°C. According to that analysis, the mass percentages of RGO are around 16.1 wt% for Co_3_O_4_ NP/RGO, 16.0 wt% for Co_3_O_4_ NR/RGO, 17.4 wt% for Co_3_O_4_ NC/RGO, and 14.0 wt% for Co_3_O_4_ OC/RGO.

### Shape-dependent ORR catalytic activity of the Co_3_O_4_ nanocrystals

**Figure 4:**
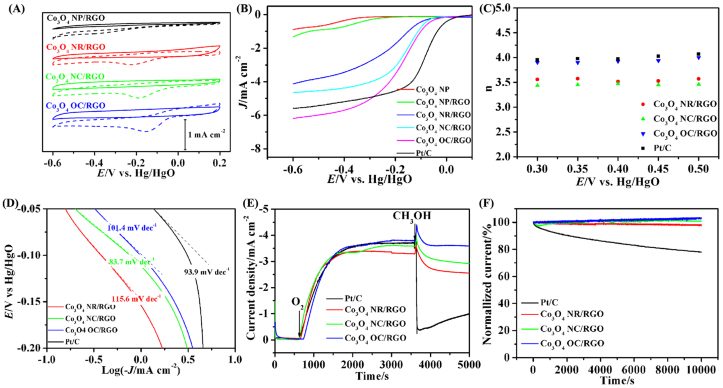
(A) CV curves of Co_3_O_4_ nanocrystals/RGO composites on glassy carbon electrodes in N_2_-saturated (solid line) or O_2_-saturated 0.1 M KOH (dash line); (B) Rotating-disk voltammograms, (C) The electron transfer number (n) profiles obtained from (B), and (D) Tafel plots for the Co_3_O_4_ /RGO composite electrodes and the commercial Pt/C electrode; (E) *J*-T chronoamperometric responses at −0.40 V versus Hg/HgO reference electrode at a rotating rate of 2400 rpm. The 0.1 M KOH solution electrolyte is firstly bubbled by N_2_ for 30 min, and then is introduced by O_2_ gas for around 3000 s, and is finally added by 20 vol% of methanol; (F) Chronoamperometric responses (percentage of current retained versus operation time) of the kept at −0.40 V versus Hg/HgO reference electrode in O_2_-saturated 0.1 M KOH electrolyte at a rotating rate of 2400 rpm.

To assess their ORR catalytic activity, the nanocrystal materials were loaded (with equal mass loading) onto glassy carbon electrodes. The electrodes were interrogated by cyclic voltammetry (CV) in O_2_- and for reference, N_2_-, saturated 0.1 M KOH solutions, and the data are shown in Fig. 4A. The Co_3_O_4_ NP (size ~10 nm) on the surface of RGO sheets exhibited very poor ORR activity: with an onset potential of ~−0.25 V vs. Hg/HgO. The Co_3_O_4_ NR/RGO hybrids with exposed surfaces dominated by {110} showed a much more positive ORR onset potential (~−0.1 V), suggesting higher ORR catalytic activity than Co_3_O_4_ NP/RGO. Remarkably, the Co_3_O_4_ NC/RGO with the six exposed {100} surfaces and the Co_3_O_4_ OC/RGO nanocomposites with the eight exposed {111} surfaces achieved even more positive onset potentials, e.g., ~−0.06 V for Co_3_O_4_ NC/RGO, and ~−0.04 V for Co_3_O_4_ OC/RGO, closely approaching that of Pt/C, the gold standard for ORR catalysts.

The ORR kinetics of the Co_3_O_4_/RGO composites was investigated using the rotating-disk electrode (RDE) technique in O_2_-saturated 0.1 M KOH electrolyte. As can be seen from the LSV curves in Figure 4B, the ORR process is diffusion controlled when the potential is negative to −0.20 V, mixed diffusion kinetic controlled in the potential region from −0.20 to −0.10 V, and kinetic controlled in the potential range from −0.01 to 0 V. Unsupported Co_3_O_4_ NP prepared by thermal decomposition of Co(CO_3_)_1/2_OH precursors, as we reported previously^26^, exhibited much lower onset potential and diffusion-limited current density than those of the present four Co_3_O_4_/RGO composite electrodes, suggesting the positive effect of RGO on the ORR catalytic activity of Co_3_O_4_^17^. Among the four Co_3_O_4_/RGO composite electrodes, the Co_3_O_4_ NP/RGO composite catalyst shows the lowest onset potential, whereas the Co_3_O_4_ OC/RGO composite shows the highest one. The half-wave potentials of the composite catalysts are in the sequence of Co_3_O_4_ OC/RGO (−0.14 V) > Co_3_O_4_ NC/RGO (−0.16 V) > Co_3_O_4 _NR/RGO (−0.20 V) > Co_3_O_4_ NP/RGO (−0.33 V). In the diffusion controlled region, the diffusion-limited current densities follow the trend of the half-wave potentials for the composite catalyst series. Together, these results suggest that the Co_3_O_4_ OC/RGO composite catalyst exhibits the highest ORR catalytic activity among the four samples, and the different catalytic activities can be attributed to the different morphologies of the Co_3_O_4_ nanocrystals in the composites.

A series of rotating disk voltammograms of oxygen reduction are shown in Figure SI-(6–9)A with the commercial Pt/C and the Co_3_O_4_/RGO composite catalysts at different rotation rates in O_2_-saturated 0.1 M KOH electrolyte. The RDE data were analyzed using the Koutecky-Levich equation (Eq. 1 in the experimental section), according to which a plot of the inverse current density *J*^−1^ versus Ω^−1/2^, shown in Figure SI-(6–9)B, should yield a straight line with the intercept corresponding to *J*_k_ and the slope reflecting the so-called B factor. The electron transfer number for the O_2_ reduction process can be calculated from the B factor according to Eq. 2 (see the experimental section). The linearity of the Koutecky-Levich plots and the near parallelism of the fitting lines are consistent with the first-order reaction kinetics with respect to the concentration of the dissolved oxygen and implicate similar electron transfer numbers for the ORR at different potentials in the region of −0.30 V to −0.50 V. The calculated electron transfer numbers (n) for the commercial Pt/C and the Co_3_O_4_/RGO composite catalysts in the potential region of −0.30 to −0.50 V are shown in Figure 4C. We can see that the Co_3_O_4_ OC/RGO composite electrode can catalyze the ORR via a 4 e process, in much the same way as a high-quality commercial Pt/C catalyst does, which is impressive for a non-Pt catalyst. However, the ORR electron transfer number for the Co_3_O_4_ NR/RGO and Co_3_O_4_ NC/RGO composite catalysts were calculated to be ~3.5, suggesting incomplete reduction of oxygen, but still domination by the 4e process.

The ORR catalytic activity of the Co_3_O_4_/RGO hybrid catalysts can also be gleaned from the Tafel slopes at low and high overpotentials in O_2_-saturated 0.1 M KOH aqueous solution. The Tafel data are shown in Figure 4D. The *E* versus log (-*J*) curves of the samples similarly show two Tafel slopes at low and high overpotentials, respectively, indicating a similar change in reaction mechanisms with the potential. The two slopes can be explained in term of the isotherms at two different O_2_ coverages; i) the Temkin isotherm (high O_2_ coverage) associated with an intermediate oxide coverage arising from ORR at low overpotential, whereby the first electron transfer step involving an adsorbed product such as OH^−^ is the rate-determining step; and ii) the Langmuir isotherm (low O_2_ coverage) at high overpotential wherein significant oxide coverage ceases to exist, which is commonly the case when a two-electron transfer reaction is the rate-determining step. This is a characteristic feature of ORR on mixed valence spinel oxide, e.g., Co-based ORR catalysts^25^,^27^. As can be observed in Figure 4D, the Co_3_O_4_ NC (83.7 mV/decade) and Co_3_O_4_ OC (101.4 mV/decade) on RGO sheets exhibit smaller Tafel slopes at the over-potentials from −0.05 V to −0.10 V than the Co_3_O_4 _NR/RGO hybrid (115.6 mV/decade) in 0.1 M KOH electrolyte, demonstrating high ORR catalytic activities close to that of the commercial Pt/C catalyst (93.9 mV/decade).

Other important performance metric for an ORR catalysts include the tolerance of the commonly used fuel molecules and cycle stability, which are especially relevant for fuel cells such as direct methanol fuel cell. To examine the possible crossover effect for the catalytic performance, we measured the electrocatalytic selectivity of the Co_3_O_4_/RGO composite electrode against electro-oxidation of methanol molecules. The current density-time (*J*-T) chronoamperometric response profiles are shown in Figure 4E. For the commercial Pt/C electrode, a sharp decrease (~85%) in current density was observed upon methanol addition (20 vol%) into the O_2_-saturated 0.1 M KOH electrolyte. However, the amperometric responses of the Co_3_O_4_/RGO composite electrodes are strong and stable, showing a retention ratio of at least 80% after the addition of methanol. Such high selectivity of the Co_3_O_4_/RGO composite electrodes toward the ORR and the remarkably good tolerance to crossover effect can be attributed to the much lower ORR potential than required for oxidation of the fuel molecules^28^. Moreover, the Co_3_O_4_/RGO hybrid electrodes also exhibited excellent stability as measured by chronoamperometric measurements (Figure 4F). At a constant voltage of −0.40 V vs Hg/HgO, the ORR current density produced in the hybrid catalysts almost had no decay over 10000 s of continuous operation, whereas the commercial Pt/C catalyst exhibited ~22% decrease in current density. Thus, in comparison with the commercial Pt/C catalyst, our Co_3_O_4_/RGO composite electrodes are more insensitive to methanol molecules, thus more resistive to poisoning by the possible methanol crossover from the anode of a fuel cell, and are more stable under operating conditions.

## Discussion

**Figure 5:**
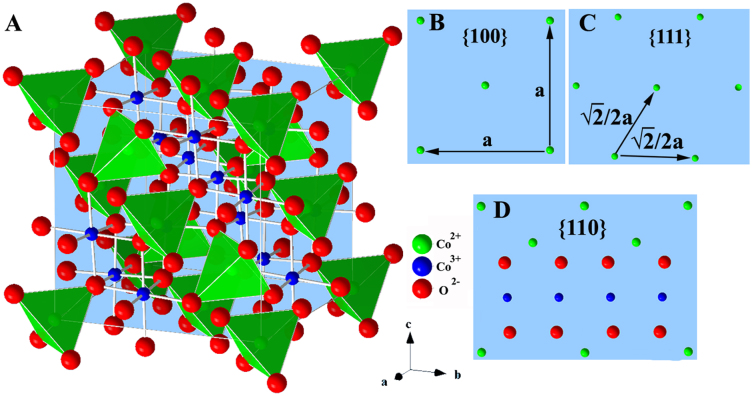
Structure models of the spinel Co_3_O_4_ nanocrystals. (A) Three-dimensional atomic arrangement and (B–D) Surface atomic configurations in the {100}, {111} and {110} planes.

Co_3_O_4_ has the normal-spinel structure Co^2+^Co_2_^3+^O_4_, in which the Co^2+^ ion in the formula unit occupies the tetrahedral site, while the two Co^3+^ ions occupy the octahedral sites^6^, as shown in Figure 5A. Figure 5B–D depict the close-packed planes of {001}, {111} and {110}, and their surface atomic configurations of the spinel-type Co_3_O_4_ crystals. Experimental and theoretical measurements have demonstrated that the three low Miller index planes ({100}, {110} and {111}) of such metallic oxide particles with a fcc structure differ not only in the surface atomic density but also in the electronic structure, geometric bonding and chemical reactivity^29^. As a result, those planes have different surface energies, following the order of γ{111} < γ{100} < γ{110}, which is closely parallel to the catalytic activities for CO and CH_4_ oxidation^12^,^13^,^30^,^31^.

For catalyzing CO oxidation, the CO molecule interacts preferably with the surface Co^3+^ cations, which is the only favorable site for CO adsorption, as confirmed both theoretically^32^ and experimentally^33^,^34^. The oxidation of the adsorbed CO then occurs by abstracting the surface oxygen that had been coordinated with the Co^3+^ cations. The partially reduced cobalt site, i.e., Co^2+^ cation with a neighboring oxygen vacancy, is re-oxidized by a gas-phase oxygen molecule back to the active Co^3+^ form. Consequently, the surface Co^3+^ cations are regarded as the active sites for CO oxidation, whereas the surface Co^2+^ cations are almost inactive^12^,^31^,^35^,^36^,^37^. It is known that in the Co_3_O_4_ crystal structure, the {001} and {111} planes contain only Co^2+^ cations, while the {110} plane is composed mainly of Co^3+^ cations (Figure 5B–D). This scenario has been proved by surface differential diffraction studies concluding that the Co^3+^ cations are present solely on the {110} plane^38^,^39^. Similarly, in our own experiment with the Co_3_O_4_ NR/RGO composite catalyst, the initial transformation temperatures for CO oxidation is 60°C, considerably lower than that with Co_3_O_4_ NC/RGO (100°C) and Co_3_O_4_ OC/RGO (120°C) (Figure SI-10). Although the catalytic activities of the Co_3_O_4_/RGO composites for CO oxidation are by no means optimized, our study suffices to conclude that the Co_3_O_4_ NRs with the predominantly {110} exposed surfaces have higher catalytic activity for CO oxidation than the Co_3_O_4_ NCs with the sole six {100} exposed surfaces and the OCs with the only eight {111} exposed surfaces, in excellent agreement with the literature reports^12^,^30^,^31^.

In sharp contrast, for ORR catalysis, the Co_3_O_4_ OC enclosed by the eight {111} facets on the RGO sheets was found to exhibit the highest catalytic activity among the four Co_3_O_4_/RGO nanocomposite catalysts we have studied in the present work, followed by Co_3_O_4_ NC/RGO, and then Co_3_O_4_ NR/RGO, with Co_3_O_4_ NP/RGO being the least active (Figure 4B). Surprisingly, this ORR catalytic activity order correlates very well with the surface Co^2+^ density order of the corresponding nanocrystals on RGO excepting the unsupported nanoparticles, namely, {111} > {100} > {110}. This strongly suggests that the surface Co^2+^ ions are the catalytically active sites for ORR. Note that the measured specific surface areas of the composites are 139.4 m^2^ g^−1^ for Co_3_O_4_ NP/RGO, 110.0 m^2^ g^−1^ for Co_3_O_4_ NR/RGO, 116.6 m^2^ g^−1^ for Co_3_O_4_ NC/RGO, and 98.9 m^2^ g^−1^ for Co_3_O_4_ OC/RGO composites (see the N_2_ adsorption isotherms in Figure SI-11). And the contents of Co_3_O_4_ in the composites are around 83.9 wt% for Co_3_O_4_ NP/RGO, 84.0 wt% for Co_3_O_4_ NR/RGO, 82.6 wt% for Co_3_O_4_ NC/RGO, and 86.0 wt% for Co_3_O_4_ OC/RGO (Figure SI-5B). The similar specific surface areas together with the similar amounts of catalysts used for the ORR testing exclude the possibility of the specific surface area being an important factor that determines the ORR catalytic activity. Thus, it is the exposed crystal planes of the Co_3_O_4_ nanocrystals that play a vital role in determining the ORR catalytic activity.

Assuming that the adsorption/desorption process of O_2_ on the catalytic active sites is involved in the rate-determined step of the ORR, the surface bonding of O_2_ to the composite catalysts should be critical. In general, O_2_ molecules with the bond length of 0.12 nm adsorbs on the catalytic active sites mainly via three modes (Griffths, Bridge, and Pauling)^40^. For the mode of Griffths, the angle of the adsorbed O_ads_…Co…O_ads _is around 36°. In comparison with the angle of O-Co-O in the bulk of Co_3_O_4 _(95.5° for O-Co^3+^-O, and 109.5° for O-Co^2+^-O), such a huge angle mismatch would induce a large intra-molecular stress, resulting in the weak adsorption of O_2_ on the catalytic active sites. In the Bridge mode (Co…(O_ads_ = O_ads_)…Co), the bond length of O_2_ molecules (~0.12 nm) fails to match the distance between the adjacent catalytically active sites (0.1956 nm for Co^3+^-O, and 0.1902 for Co^2+^-O). Thus we are left with the possibility that O_2_ molecules be preferably adsorbed on the catalytically active sites via the Pauling mode (Co…(O_ads_ = O)). Conceivably, when O_2_ molecules are absorbed on the catalytically active sites via the Pauling mode, it is the surface Co^2+^ (3d^5^4s^2^) cations rather than the surface Co^3+^ (3d^5^4s^1^) cations which prefer to transfer electrons to the absorbed O_2_ molecules to weaken and to assist breaking the O-O bond, meanwhile leaving themselves oxidized to Co^3+^. This suggests that the surface Co^2+^ sites should be the catalytically active sites instead of Co^3+^ sites for ORR, and can naturally explain why the Co_3_O_4_ nanocrystals with the predominant {111} and {110} exposed surfaces exhibited higher catalytic activity for ORR than that with the {110} exposed surfaces. Such explanation also applies to the observation that CoO exhibits better catalytic ORR performance than Co_3_O_4_^16^,^41^. In addition, the density of Co^2+^ cations in {111} planes (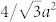) is higher than that in {100} planes (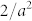), resulting in catalytic activity of the Co_3_O_4_ OC/RGO composite catalysts enclosed by the eight {111} facets than that of the Co_3_O_4_ NC/RGO composite catalyst surrounded by the six {100} exposed surfaces.

It is widely known that the RGO can serve as both a supporter for the catalyst dispersion and a conduction path for shuttling electrons involved in redox reactions. Here it is clearly the case as well, and this would indiscriminately enhance the catalytic activity of the different nanocomposite catalysts in our study. It is also possible, however, that due to the specific interaction of the different crystal faces with the RGO, the catalytic activity enhancement may be different for different nanocomposites. In particular, the interaction of the Co^2+^-rich surface with the RGO may be more beneficial to the ORR catalysis. Indeed, some studies along thins line have appeared in recent years^16^,^17^. Nevertheless, this aspect still underlines the role of the nanocrystal surfaces in ORR catalysis and adds to source of inspiration for tuning nanocrystal morphologies for optimizing catalytic efficiency.

In sum, we have demonstrated the morphological control of Co_3_O_4_ nanocrystals uniformly immobilized in situ on RGO sheets by judiciously choosing the oxidant and tuning the reaction conditions such as pH value. The resulting nanorods predominantly exposing the {110} surfaces, nanocubes surrounded by the six {100} facets, and nano-octahedrons enclosed by the eight {111} facets have allowed us to further investigate the crystal face effects on the ORR catalytic activity. We found that, while the surface Co^3+^ ions are the catalytically active sites for CO oxidation, and the surface Co^2+^ ions act as the catalytically active sites for ORR. Additionally, the density of the catalytically active sites on the surface is closely related to the catalytic activity. Accordingly, we have established that the catalytic activity for ORR of these crystalline facets decreases in the sequence of {111} > {100} ≫ {110}. This fundamental understanding shows that morphological control of metal oxide catalysts is a promising surface engineering strategy for the development of nanostructured catalysts in general and non-precious metal free nano-catalysts in particular for ORR in alkaline media.

## Methods

### Materials and reagents

Graphite flake (natural, ~325 mesh, Alfa Aesar), potassium permanganate (KMnO_4_, Riedel-de Haën), hydrogen peroxide solution (30 wt%, H_2_O_2_, BDH), nickel chloride hexahydrate (NiCl_2_·6H_2_O, Fisher), cobalt chloride hexahydrate (CoCl_2_·6H_2_O), sodium hydrogen carbonate (NaHCO_3_, BDH), ammonia water (28 ~ 29 wt%) and hydrazine monohydrate (min 98.0 wt%, N_2_H_4_·H_2_O, Wako) were used without further purification.

### Synthesis of graphene oxide (GO) sheets

Graphene oxide sheets were synthesized from natural graphite by a modified Hummers method^42^. Briefly, 0.5 g of graphite (~325 mesh, Alfa Aesar) and NaNO_3_ (0.5 g; Aldrich, >99%) were dispersed into concentrated H_2_SO_4_ (20 mL; Fisher Scientific, 98%) with an ice bath. Under vigorous stirring, KMnO_4_ (2.0 g; Riedel-de Haën, >99%) was then added gradually. After removing the ice bath, the mixture was stirred at room temperature for 24 h. As the reaction progressed, the mixture became pasty with a brownish color. Successively, 20 mL of H_2_O was slowly added to the pasty mixture while keeping the mixture in an ice bath, since the addition of water into the concentrated H_2_SO_4_ medium will release a large amount of heat. After dilution with 40 mL of H_2_O, 5 mL of 30% H_2_O_2_ (VMR) was added to the mixture, accompanied by bubbling and changing to brilliant yellow color. After continuously stirring for 2 h, the mixture was filtered and washed with DI water. Then, the products were dispersed in 10 wt% HCl aqueous solution, and washed with DI water again three times to remove impurity ions. Finally, the products were dispersed in DI water via ultrasonication, and then centrifuged at 7000 rpm for 1 h. The supernatant was collected as a GO aqueous solution with a concentration of ~1 mg mL^−1^.

### Controllable synthesis of differently shaped Co_3_O_4 _nanocrystals on reduced graphene oxide (RGO) sheets

To start with, 20 mM of CoCl_2_·6H_2_O and 40 mM of NaHCO_3_ were dissolved into 90 mL of DI water and mixed with 10 mL of GO solution, and the mixture was flushed with gaseous CO_2_ for 2 h forming Solution A. Solution A was stirred at room temperature for 12 h, and subsequently refluxed at 100°C for 10.0 h after adding in 0.1 mL N_2_H_4_, forming Solution B. The precipitates in Solution B were centrifuged and washed with DI water three times, and then freeze dried. Finally, the products were thermally treated at 400°C for 1 h in a N_2_ atmosphere with a heating rate of 5°C/min, to form the Co_3_O_4_ nanoparticles/RGO (Co_3_O_4_ NP/RGO) composites. In parallel experiments, when Solution B was poured into a 70 mL capacity autoclave with Teflon liner and then hydrothermally treated at 100°C for 12 h before the thermal treatment process, the as-formed products were labeled as Co_3_O_4_ nanorods/RGO (Co_3_O_4_ NR/RGO) composites. When Solution B and 5.0 mL of 30 wt% hydrogen dioxide (H_2_O_2_) were poured into a 70 mL capacity autoclave with Teflon liner and then hydrothermally treated at 100°C for 12 h before the thermal treatment process, the final products were labeled as Co_3_O_4_ octahedrons/RGO (Co_3_O_4_ OC/RGO) composites.

As for Co_3_O_4_ NC on RGO sheets, a typical synthesis is as follows. First, 1 mmol CoCl_2_·6H_2_O was dissolved in a mixture of 1 mL of 30 wt% hydrogen peroxide (H_2_O_2_) and 40 mL of distilled water. When the solution was clarified, the solution was maintained at pH 9.0 by adding ammonia solution (25 ~ 28 wt%). Then 5 mL of GO solution was added into the above solution, followed by stirring for 1 h. The reaction mixture was then charged into a 70 mL capacity autoclave with Teflon liner, which was then kept at 180°C for 12 h. After the reaction was completed, the autoclave was allowed to cool down to room temperature naturally and opened for product collection. The precipitates were washed with DI water three times and freeze dried. Finally, the products were thermally treated at 400°C for 1 h in a N_2_ atmosphere with a heating rate of 5°C min^−1^, to form the Co_3_O_4_ nanocubes/RGO (Co_3_O_4_ NC/RGO) composites.

### General Materials Characterization

The product morphologies were directly examined by scanning electron microscopy (SEM) using JEOL JSM-6700F at an accelerating voltage of 5 kV. Transmission electron microscopy (TEM) observations were carried out on a JEOL 2010 microscope operating both at 200 kV. X-ray diffraction (XRD) was performed on a Philips PW-1830 X-ray diffractometer with Cu kα irradiation (λ = 1.5406 Å). The step size and scan rate are set as 0.05° and 0.025°/s, respectively. X-ray photoelectron spectroscopy (XPS) was measured on a Perkin-Elmer model PHI 5600 XPS system with a resolution of 0.3–0.5 eV from a monochromated aluminum anode X-ray source with Kα radiation (1486.6 eV). The thermogravimetric analysis (TGA) was performed from 30 to 700°C on a TGA Q5000 (TA Instruments Ltd) at a heating rate of 5°C min^−1^ under an air flow of 25 mL min^−1^. Brunauer-Emmett-Teller (BET) surface areas were measured on a Coulter SA 3100 surface area analyzer.

### Catalytic measurements for oxygen reduction reaction (ORR)

Electrochemical measurements were carried out by cyclic voltammetry (CV) on a CHI 660D electrochemical workstation. A conventional, three-electrode cell consisting of glassy carbon electrode (GCE) with an area of 0.125 cm^2^ was used as the working electrode, Pt foil was employed as the counter electrode and Hg/HgO (1.0 M KOH) (MMO, 0.098 V vs. SHE) was used as the reference electrode. The working electrode was modified with a catalyst layer by dropping a suitable amount of catalyst ink on the GCE. The catalyst ink was prepared by ultrasonically dispersing 10 mg of the carbon supported catalysts in a 2.0 mL solution (1.9 mL of ethanol and 0.1 mL of 5 wt% Nafion solution) for 30 min to obtain a homogeneous solution. 10 μL of the dispersion was pipetted out and dropped onto a glassy carbon rotating disk electrode of 3 mm in diameter, which was then dried in air. CV experiments were conducted at room temperature in 0.1 M KOH solution saturated with nitrogen. For all of the experiments, stable voltammogram curves were recorded after scanning for 20 cycles in the potential region from 0 to 0.6 V in 0.1 M KOH solution. Polarization curves for the oxygen reduction reaction (ORR) were obtained in 0.1 M KOH solution using the rotating ring disk electrode (RRDE-3A). Before the RRDE study, the electrodes were cycled at 50 mV s^−1^ between 0 and 0.6 V until reproducible cyclic voltammograms were obtained. Normalized currents are given in terms of geometric weight (mA cm^−2^). The working electrode was scanned cathodically at a rate of 5 mV s^−1^ with varying rotating speed from 400 rpm to 2400 rpm. Koutecky-Levich plots (*J*^−1^ vs. ω^−1/2^) were analyzed at various electrode potentials. The slopes of their best linear fit lines were used to calculate the number of electrons transferred (n) on the basis of the Koutecky-Levich equation: 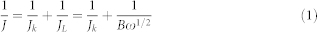

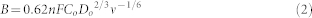

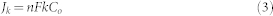
Where *J* is the measured current density, *J*_k_ and *J*_L_ are the kinetic- and diffusion-limiting current densities, ω is the angular velocity, n is transferred electron number, F is the Faraday constant (96485 C mol^−1^), C_o_ is the bulk concentration of O_2_ (1.2 × 10^−6^ mol cm^−3^), ν is the kinematic viscosity of the electrolyte (0.01 cm^2^ s^−1^), *D*_o_ is the O_2_ diffusion coefficient (1.9 × 10^−5^ cm^2^ s^−1^), and *k* is the electron-transfer rate constant.

### Catalytic measurements for CO oxidation

The catalytic activity toward CO oxidation was evaluated in a continuous flow reactor. In brief, the reaction gas, 5% CO in nitrogen (99.999%) (10 mL min^−1^) and air (99.999%) (40 mL min^−1^) was fed to a catalyst (22.5 mg) containing fixed-bed flow reactor made of glass with an inner diameter of 2.4 mm. Steady-state catalytic activity was measured at each chosen temperature, from room temperature to 200°C in a step of 20°C. The effluent gas was analyzed on-line by an on-stream gas chromatograph (Ramiin GC 2060) equipped with a TDX-01 column.

## Author Contributions

S.Y. designed the experiments. J.X. carried out the experiments. Q.K. carried out the CO oxidation experiment. J.X. and S.Y. analyzed the data and wrote the manuscript. Q.K., F.X., S.W. and L.G. contributed to the data analysis. All the authors discussed the research.

## Supplementary Material

Supplementary Information Supplementary information
