# Supplementary material for: Surface Structure Dependent Electrocatalytic Activity of Co3O4 Anchored on Graphene Sheets toward Oxygen Reduction Reaction
Source: Sci Rep. 2013 Jul 29;3:2300. doi: 10.1038/srep02300 (PMC3725507; doi:10.1038/srep02300)
Supplement: Supplementary Information [file srep02300-s1.doc]

[Supplementary information](javascript:;)

Surface Structure Dependent Electrocatalytic Activity of Co_3_O_4_ Anchored on Graphene Sheets toward Oxygen Reduction Reaction

Junwu Xiao,^1,2^* Qin Kuang,^1^ Shihe Yang,^1*^ Fei Xiao,^2^ Shuai Wang^2^

^1^Department of Chemistry, William Mong Institute of Nano Science and Technology, The Hong Kong University of Science and Technology, Clear Water Bay, Kowloon, Hong Kong

^2^Department of Chemistry and Chemical Engineering, Hubei Key Laboratory of Material Chemistry and Service Failure, Key Laboratory for Large-Format Battery Materials and System, Ministry of Education, Huazhong University of Science & Technology, Wuhan, PR China

E-mail: [chjwxiao@hust.edu.cn](mailto:chjwxiao@hust.edu.cn), [chsyang@ust.hk](mailto:chsyang@ust.hk)

**
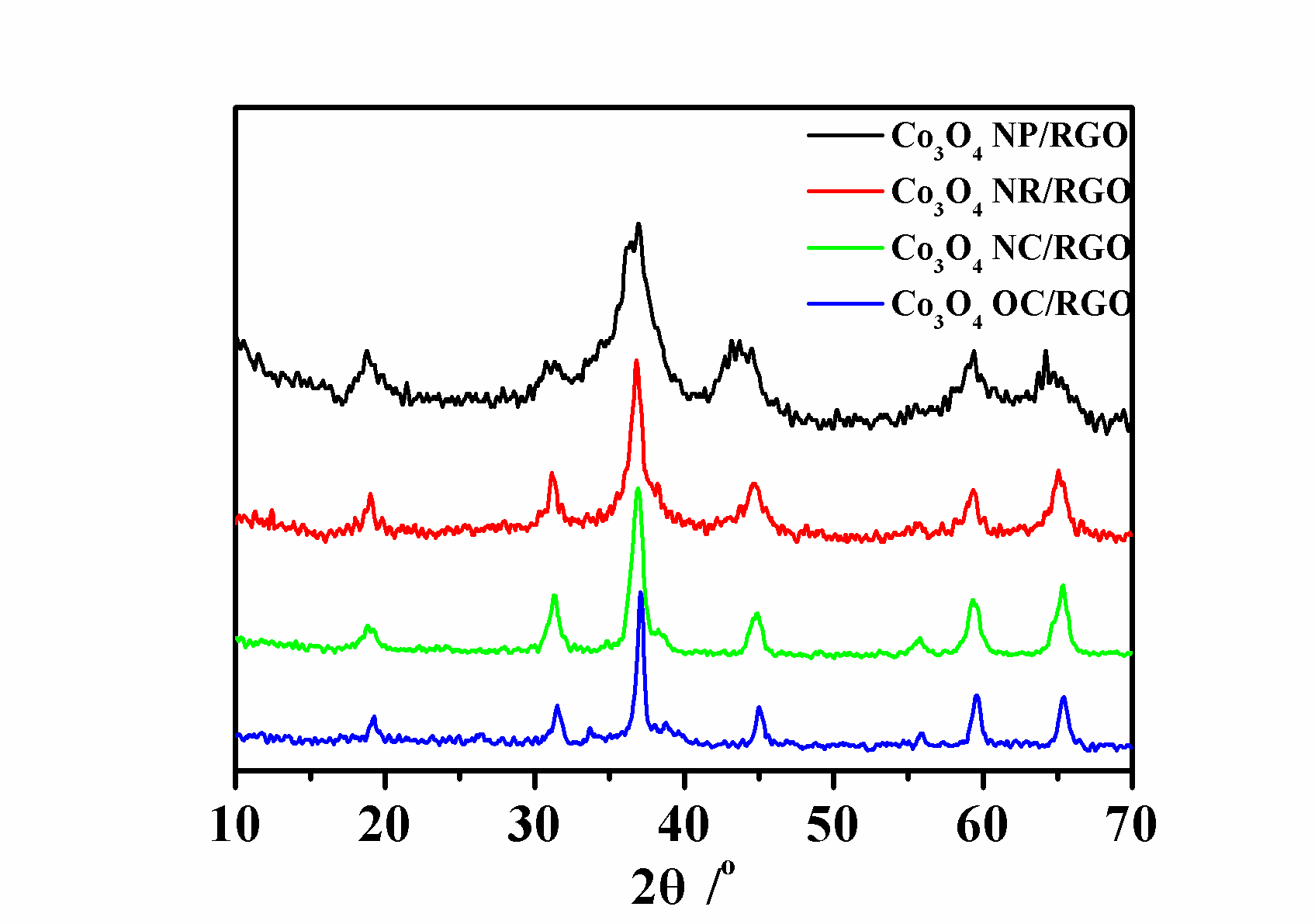
**

**Figure SI-1.** XRD patterns of Co_3_O_4_ nanoparticles (NP), nanorods (NR), nanocubes (NC), and octahedron (OC) anchored on the surface of RGO sheets, in accordance with the standard pattern of Co3O4 (JCPDS 65-3103).

**
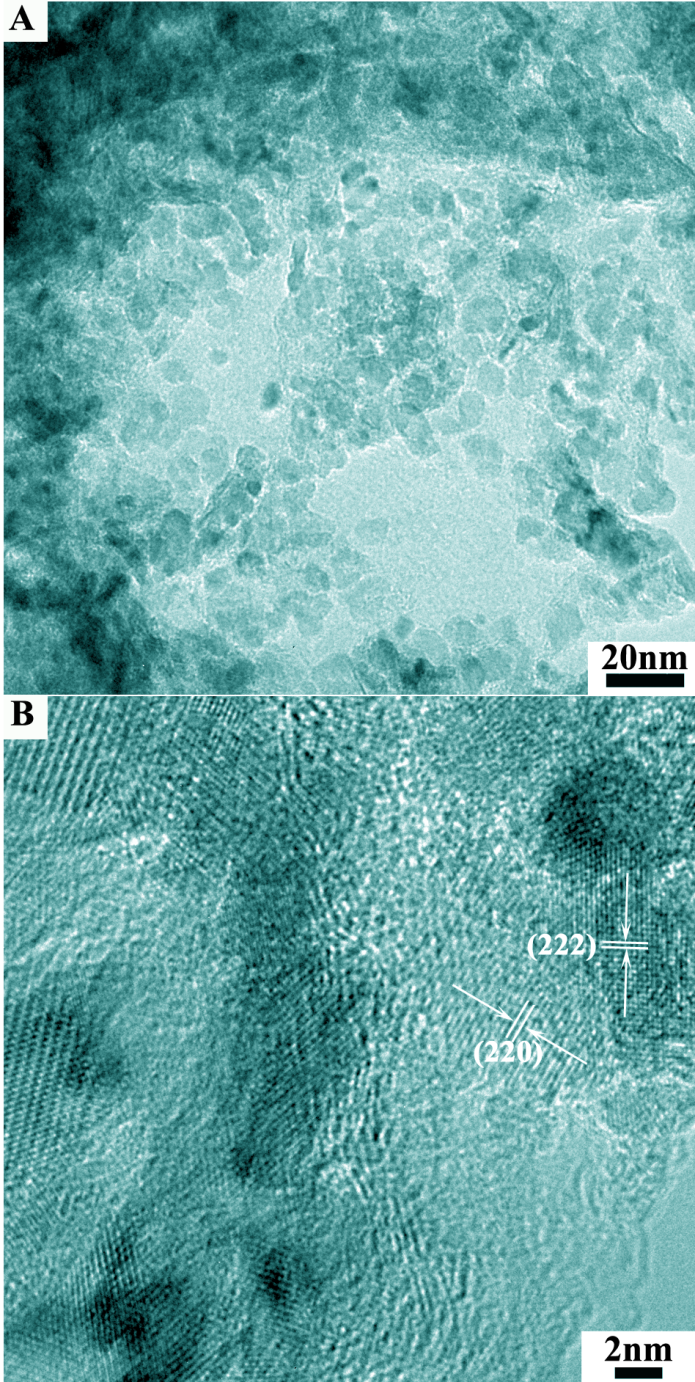
**

**Figure SI-2. (A)** TEM and (B) high resolution TEM (HRTEM) images of Co_3_O_4_ nanoparticles grown on the surface of RGO sheets, which are calcined from the precursors.

**
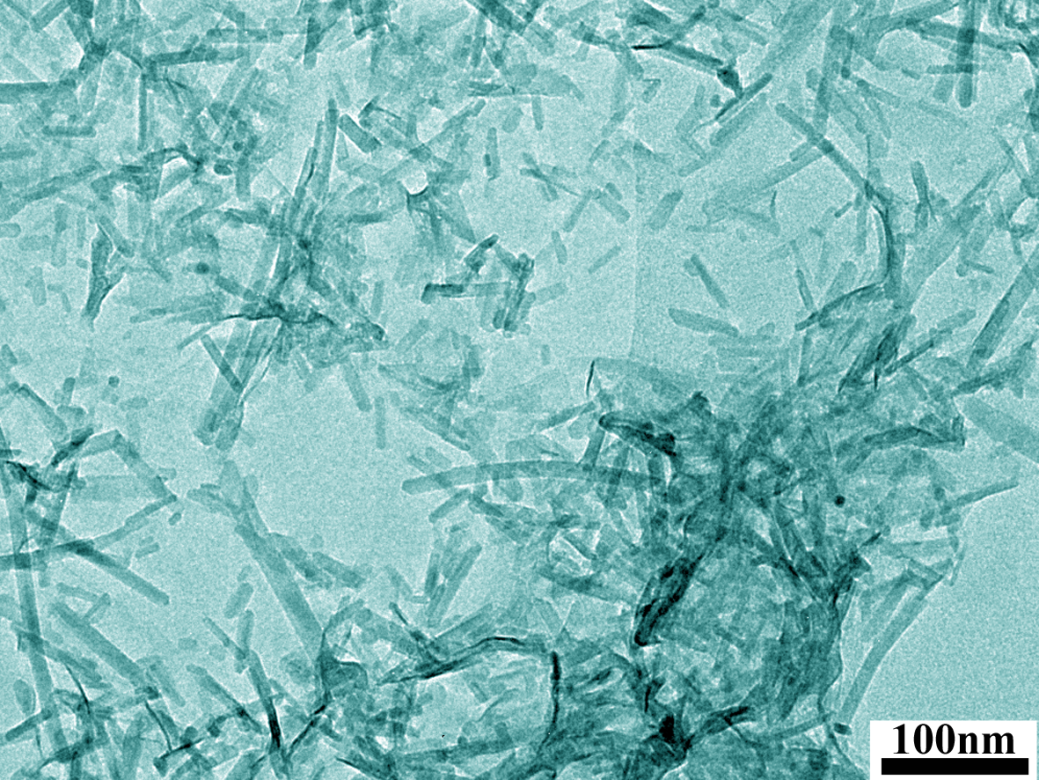
**

**Figure SI-3.** TEM image of cobalt carbonate hydroxide (Co(CO_3_)0.5OH•0.11H_2_O) nanorods grown on the surface of graphene sheets.

**
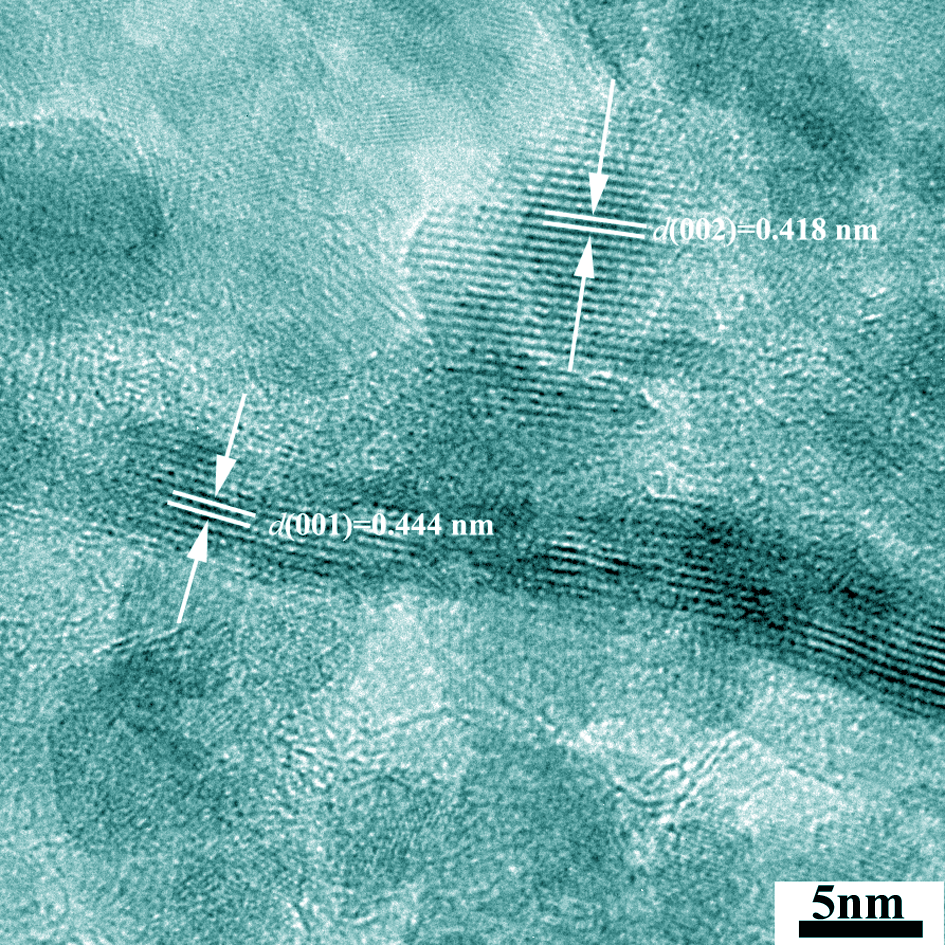
**

**Figure SI-4.** HRTEM image of the samples obtained at 100 ^o^C for 3 h.

**
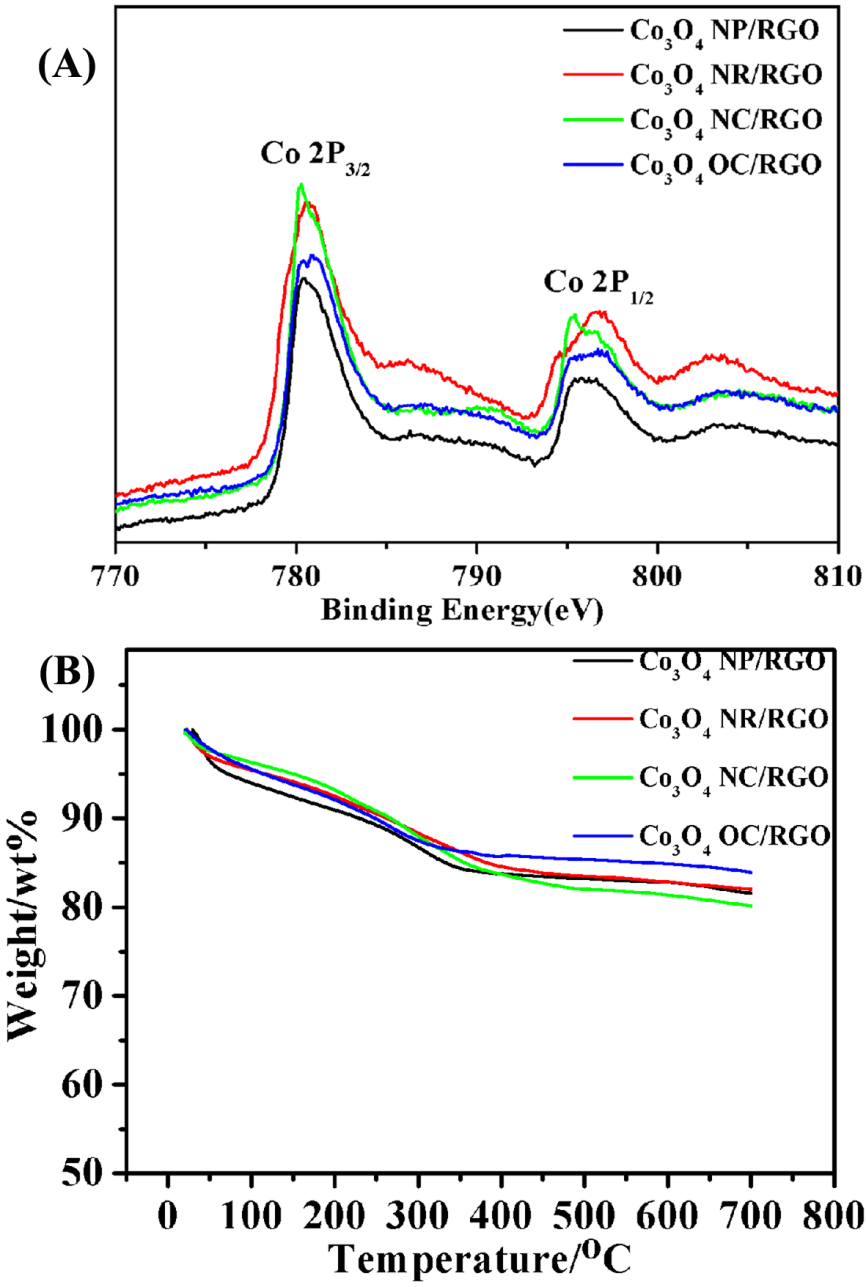
**

**Figure SI-5.** XPS spectra (A) and XRD patterns (B) of Co_3_O_4_ nanoparticles (NP), nanorods (NR), nanocubes (NC), octahedron (OC) anchored on the surface of RGO sheets.

**A**

**ω^-1/2^/rad^-1/2^ s^1/2^**

**B**

**Figure SI-6.** Rotating disk voltammograms at different rotation rates and corresponding Koutecky-Levich plots at different electrode potentials of the commercial Pt/C catalyst in O_2_-saturated 0.1 M KOH electrolyte.

**
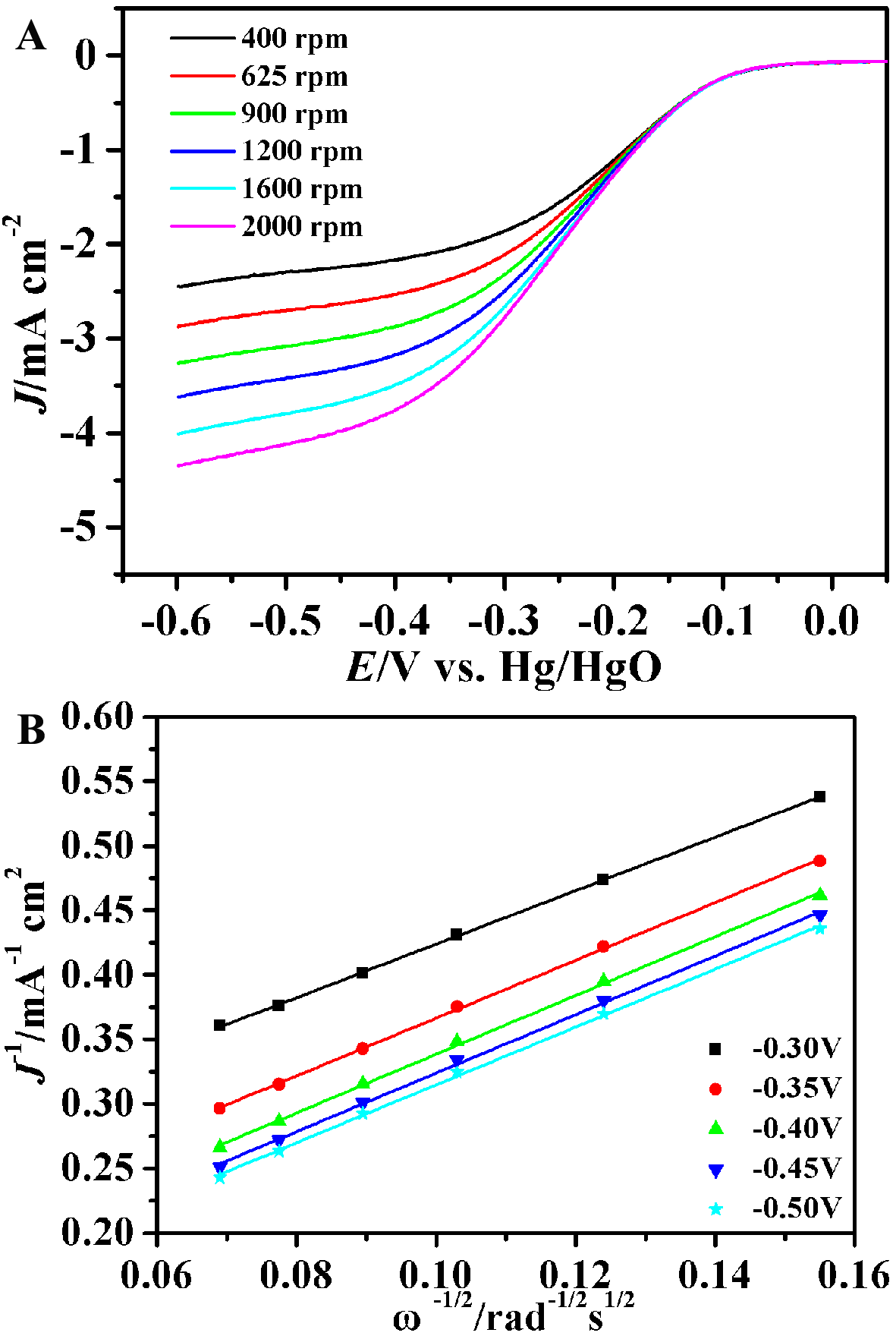
**

**Figure SI-7.** Rotating disk voltammograms at different rotation rates and corresponding Koutecky-Levich plots at different electrode potentials of the Co_3_O_4_ NR/RGO composite electrode in O_2_-saturated 0.1 M KOH electrolyte.

**
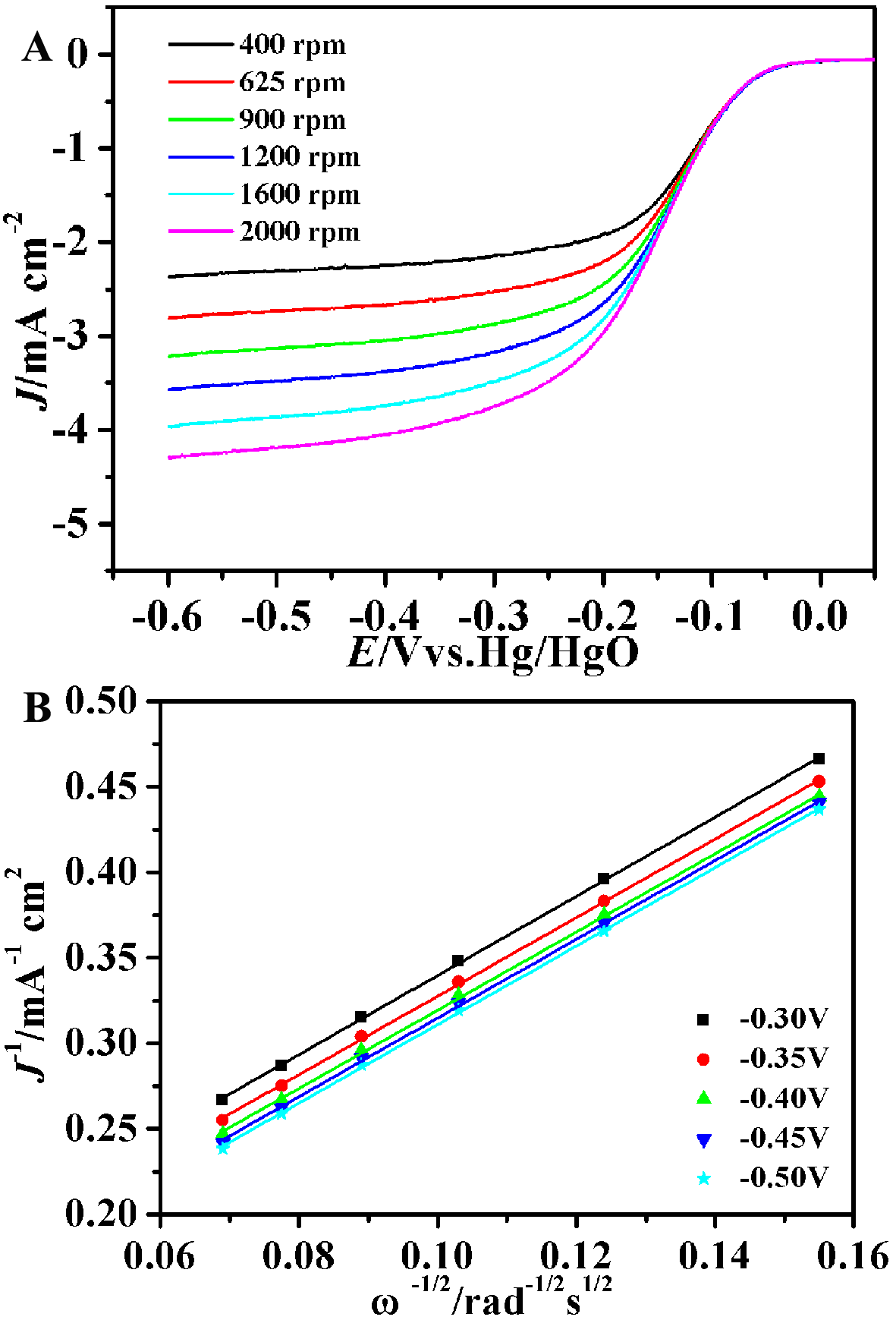
**

**Figure SI-8.** Rotating disk voltammograms at different rotation rates and corresponding Koutecky-Levich plots at different electrode potentials of the Co_3_O_4_ NC/RGO composite electrode in O_2_-saturated 0.1 M KOH electrolyte.

**
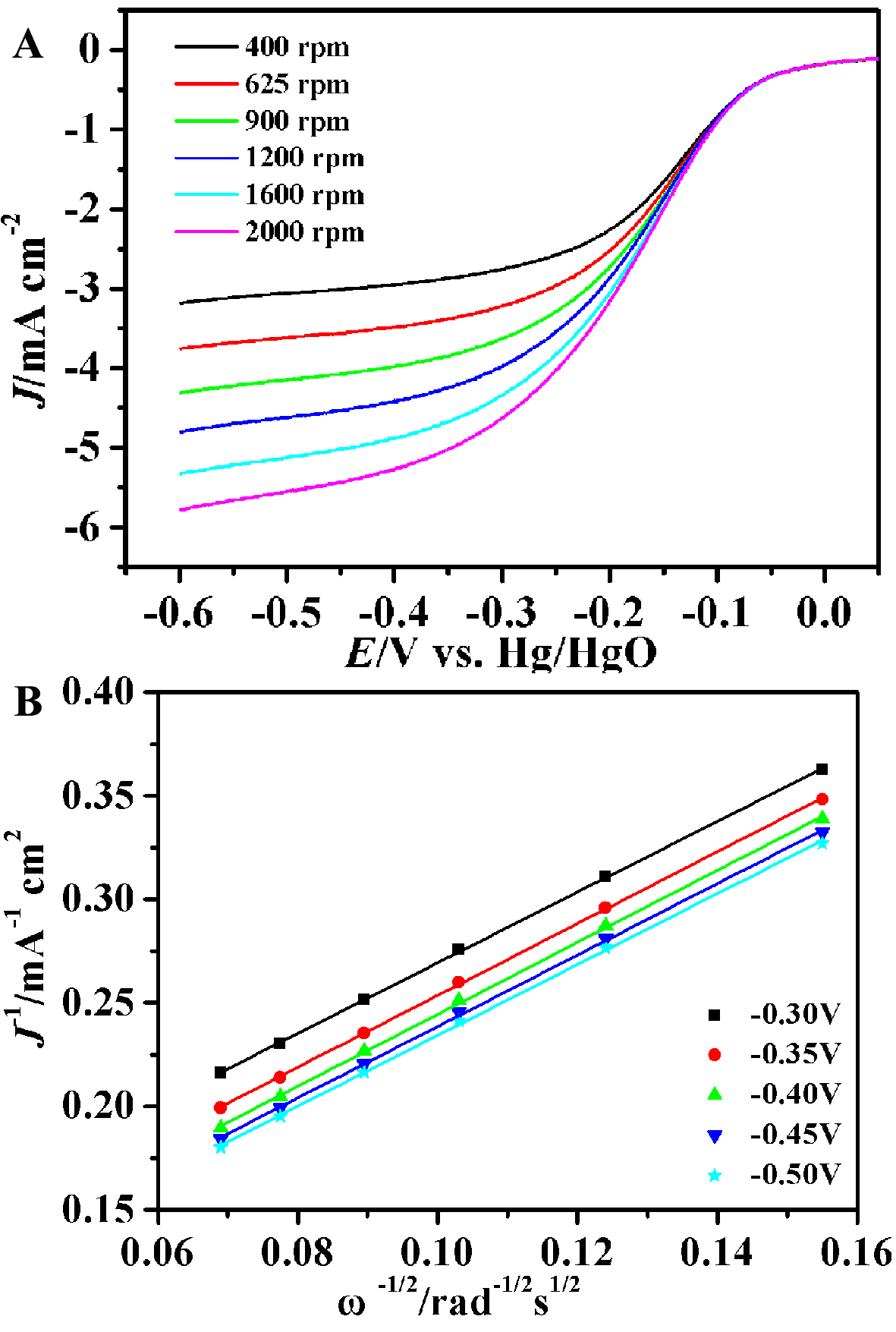
**

**Figure SI-9.** Rotating disk voltammograms at different rotation rates and corresponding Koutecky-Levich plots at different electrode potentials of the Co_3_O_4_ OC/RGO composite electrode in O_2_-saturated 0.1 M KOH electrolyte.

**
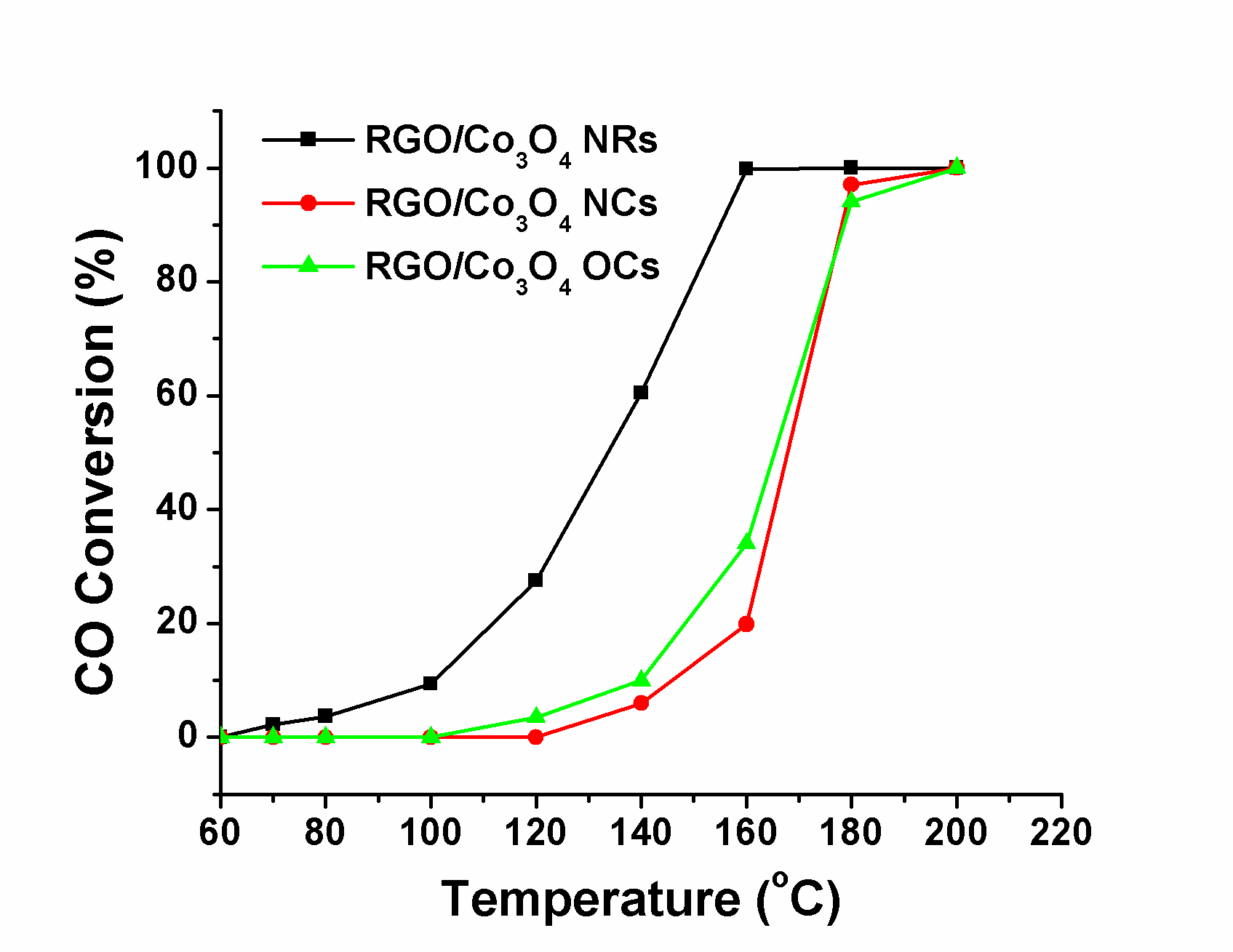
**

**Figure SI-10.** The catalytic properties of the RGO/Co_3_O_4_ nanorods (NRs), RGO/Co_3_O_4_ nanocubes (NCs), and RGO/Co_3_O_4_ octaohedraons (OCs) composites, i.e., the catalytic oxidation of CO, as a function of temperature.


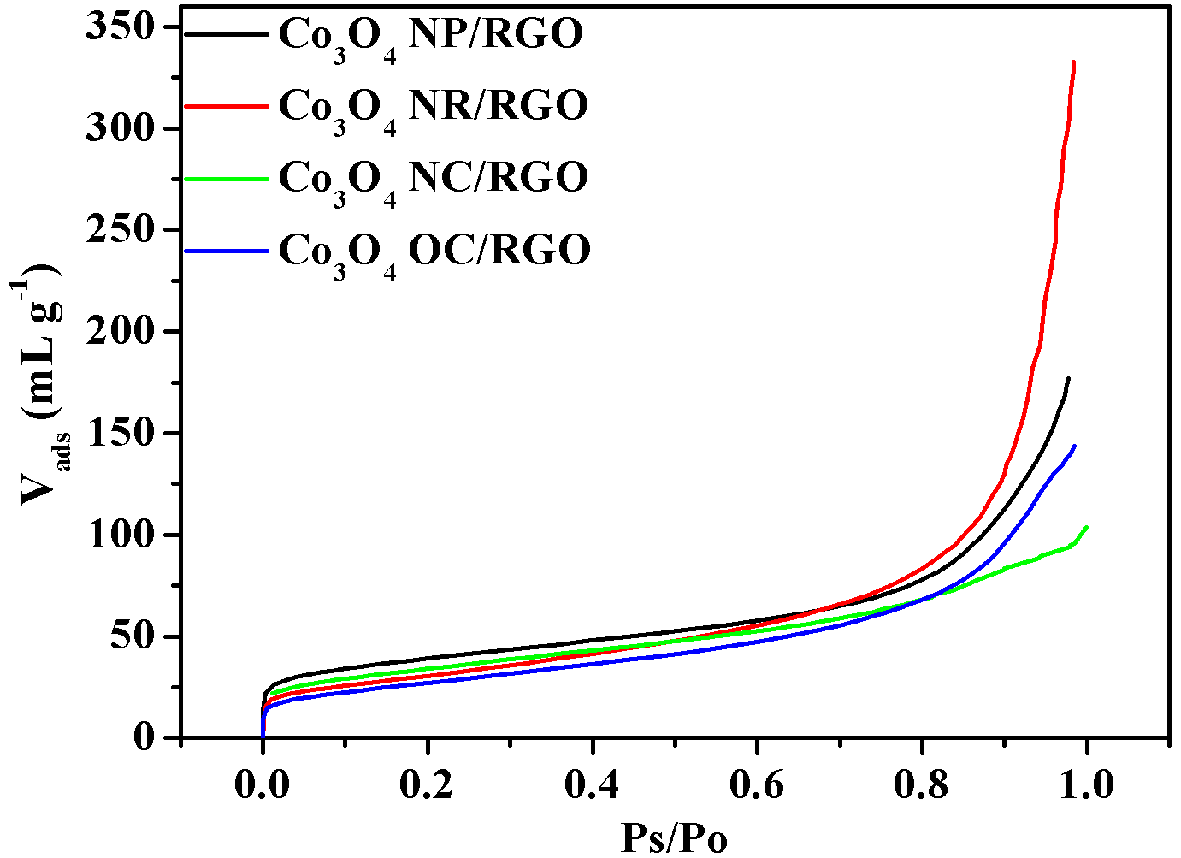


**Figure SI-11.** N_2_ adsorption isotherms of the Co_3_O_4_ nanoparticle (NP)/RGO, Co_3_O_4_ nanorod (NR)/RGO, Co_3_O_4_ nanocube (NC)/RGO, and Co_3_O_4_ nano-octahedron (OC)/RGO composites measured at standard temperature and pressure.
